# Supplementary material for: Splice-Junction-Based Mapping of Alternative Isoforms in the Human Proteome
Source: Cell Rep. Author manuscript; Available in PMC 2020 Jan 15. (PMC6961840; doi:10.1016/j.celrep.2019.11.026)
Supplement: 2 [file NIHMS1546469-supplement-2.zip › DF1/PXD009737/TestisTrypsin/TestisTrypsin_47_TTLL7_SGAMIQGPSPLDLNTELPYQSTMKR.pdf]

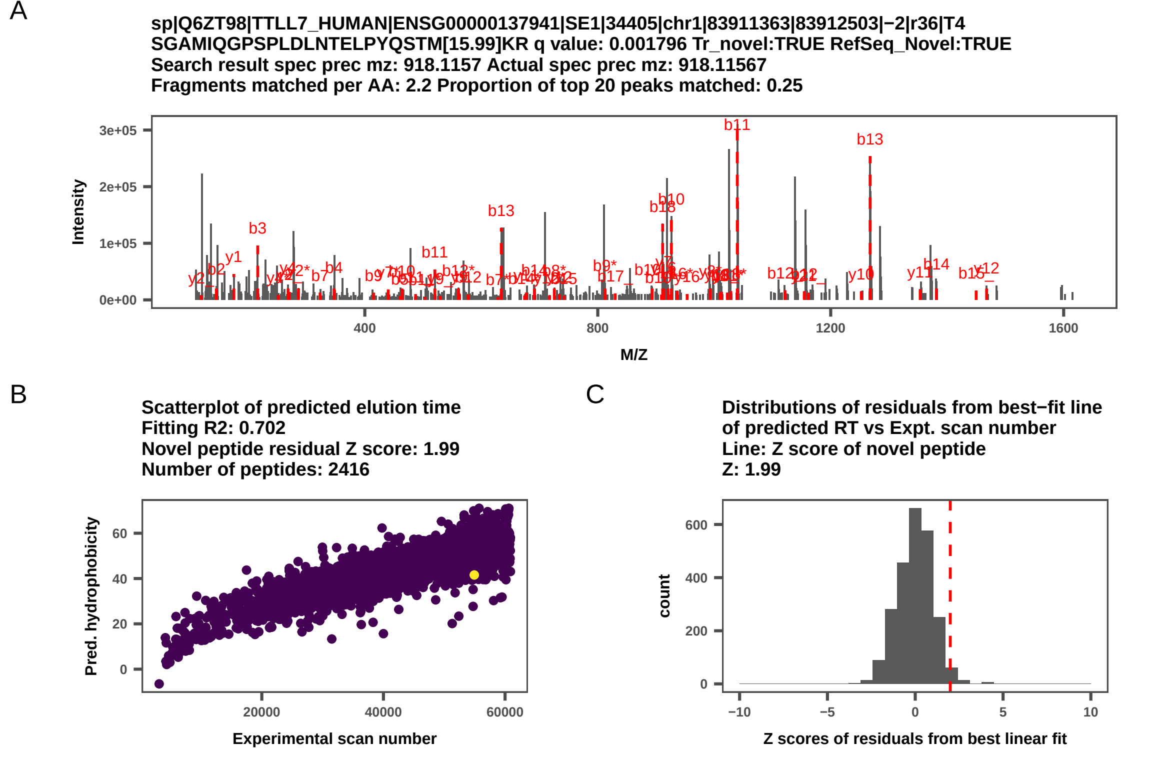

A
sp|Q6ZT98|TTLL7_HUMAN|ENSG00000137941|SE1|34405|chr1|83911363|83912503|−2|r36|T4
SGAMIQGPSPLDLNTELPYQSTM[15.99]KR q value: 0.001796 Tr_novel:TRUE RefSeq_Novel:TRUE
Search result spec prec mz: 918.1157 Actual spec prec mz: 918.11567
Fragments matched per AA: 2.2 Proportion of top 20 peaks matched: 0.25
Intensity
3e+05
2e+05
1e+05
0e+00
M/Z
400
800
1200
1600
b11
b13
b13
b3
y1
b2
b11
b10
b18
b14
y11
y10
y12
B
Scatterplot of predicted elution time
Fitting R2: 0.702
Novel peptide residual Z score: 1.99
Number of peptides: 2416
Pred. hydrophobicity
60
40
20
0
20000
40000
60000
Experimental scan number
C
Distributions of residuals from best−fit line
of predicted RT vs Expt. scan number
Line: Z score of novel peptide
Z: 1.99
count
600
400
200
0
−10
−5
0
5
10
Z scores of residuals from best linear fit
